# Impact of Drying Method on the Evaluation of Fatty Acids and Their Derived Volatile Compounds in ‘Thompson Seedless’ Raisins

**DOI:** 10.3390/molecules25030608

**Published:** 2020-01-30

**Authors:** Dong Wang, Hafiz Umer Javed, Ying Shi, Safina Naz, Sajid Ali, Chang-Qing Duan

**Affiliations:** 1 Center for Viticulture & Enology, College of Food Science and Nutritional Engineering, China Agricultural University, Beijing 100083, China; wangdong@cau.edu.cn (D.W.); qaziumerjaved@cau.edu.cn (H.U.J.); shiy@cau.edu.cn (Y.S.); 2 Beijing Food Industrial Research Institute, Beijing 100075, China; 3 Beijing Industrial Technology Research Institute, Beijing 101111, China; 4 Key Laboratory of Viticulture and Enology, Ministry of Agriculture, Beijing 100083, China; 5 Department of Plant Sciences, School of Agriculture and Biology, Shanghai Jiao Tong University, 800 Dongchuan Road, Minhang District, Shanghai 200240, China; 6 Department of Horticulture, Faculty of Agricultural Sciences and Technology, Bahauddin Zakariya University, Multan 60800, Pakistan; safinz ch.sajid15@yahoo.com (S.A.)

**Keywords:** fatty acids, UFAO-derived compounds, air- and sun-drying, raisins, GC/MS

## Abstract

Air- and sun-dried raisins from Thompson Seedless (TS) grapes were analyzed under GC/MS to evaluate fatty acids (FAs) and their derived volatile compounds, coming from unsaturated fatty acids oxidation. A total of 16 FAs were identified in TS raisins, including 10 saturated fatty acids (SFAs) and 6 unsaturated fatty acids (USFAs). The contents of C18:0, C15:0, and C16:0 among SFAs and C18:3, C18:2 and C18:1 in USFAs were significantly higher. Furthermore, USFAs such as C16:1 and C20:1 were only identified in air-dried raisins. The principal component analysis showed the increased content of FAs and FA-derived compounds were in air-dried and sun-dried raisins, respectively. Among FA-derived compounds, 2-pentyl furan, 3-octen-2-one, 1-hexanol and heptanoic acid were more potent. This study shows that air-drying is more favorable for the production of fatty acids (SFAs and USFAs), whereas sun-drying is more advantageous in terms of fatty acid-derived volatiles.

## 1. Introduction

Raisin is a dried grape blessed with a particularly appealing sweet taste, and nutritional and energy value. A wide variety of raisins are being used around the world either as fruits, brewing or in cultural cuisines as dessert. According to the United States Department of Agriculture [1], China is a major producer of raisins (1.90 million tons per year), and raisins have an important role in the economy. The global raisin production is about 1.25 million metric tons, with China ranked third following America and Turkey in the year 2016–17 [2]. About 20 kinds of grape varieties are cultivated for raisin production. Among these, TS is a leading grape variety and is consumed as fresh grapes, raisins, and wines. The ‘TS’ is a versatile variety because it can be used as a green raisin (sun-dried) or golden raisin (commercial dehydrator). The relatively high sugar content is the dominant feature of ‘TS’ variety, which accounts for 85–90% of the acreage in the populated raisin-growing region ‘Turpan’, in China [2].

Traditionally, raisins have been produced from fully ripened grapes by air- or sun-drying methods, or by using prevailing technologies such as microwave, freezing, and oven drying [3]. Various drying methods have been introduced for raisin production, but air-drying has gained more popularity than others in Turpan (China). The ultimate purpose of any drying technique is to produce top-quality raisins at a low price. The air-drying method may significantly affect fatty acids and their volatile compounds in comparison with the sun-drying process. The direct comparison of both drying methods with respect to fatty acid composition and their derived compounds has generally been overlooked so far. 

The aroma is the most striking feature of consumer preferences. Raisin flavor generally increased and changed, when they are being used as an ingredient of processed food. In recent years, a large number of volatiles affecting the aroma of raisins have been identified. It has already been reported that raisin volatile compounds are produced due to fresh grapes, glycosidic binding, Maillard reaction, unsaturated fatty acids, and carotenoids [4]. FA-derived volatile compounds were the major contributor to raisin aroma [4,5,6], which mainly consists of six-carbon (C_6_) and nine-carbon (C_9_) compounds of aldehydes, alcohols, and esters. These volatiles have also been known as a major source of aroma in grapes and wine [7], and are produced by the oxidative breakdown of USFAs. This process takes place through the autoxidation of USFAs [8] or the lipoxygenase (LOX) hydroperoxide lyase (HPL) pathway, in which the major enzyme such as alcohol dehydrogenases (ADHs), LOX and HPL play an important role in volatile production [9,10].

The oxidative degradation of USFAs produces the volatile compounds, and is also a key concern of food scientists and consumers because it significantly influences sensory quality, nutritional value, and the shelf life of the product. The production of a large number of compounds, such as (*E*)-2-nonenal, (*E,E*)-2,4-nonadienal, (*E*)-2-octenal, heptanal, (*E*)-2-heptenal and (*E*)-2-hexenal, has already been reported by the oxidation of linoleic acid whereas decanal and octanal are produced by the oxidation of oleic acid. Pentanoic acid may originate from the degradation of SFAs [11,12]. In addition, the compounds derived from USFAs include ester, alcohol, and aldehydes, and these compounds play a pivotal role in the generation of fruity, floral and green leafy aroma in raisins.

Storage causes a deterioration in the quality, freshness, and aroma of foodstuffs, which can easily be recognized by the consumer [13]. During storage, the autoxidation of fatty acids, in addition to the formation of volatile compounds, also causes rancidity. Packaging materials fulfill several purposes, including reduction of rancidity [14], protection against adulteration, and maintenance of the anticipated quality, aroma and flavor of the product [15]. Likewise, for consumer acceptance, the aroma and flavor should be maintained during raisin storage. The concentration and composition of FAs and their derived volatile compounds produced in the air-drying raisins may differ from those of sun-drying. Therefore, the aim of our research work was to investigate the generation and change the regularity of FA and volatile compounds in TS raisin during storage.

## 2. Results

FAs are an essential component of our body that we usually take from our foods. Raisin is one of the main sources of FAs. Furthermore, their derived compounds produce different kinds of aromas (fatty, roasted, fruity, etc.). FAs and their derived compounds can be released or generated under the influence of air- and sun-drying methods during storage.

### 2.1. Fatty Acid Composition

The compositions of 16 FAs were obtained from TS raisins (air- and sun-dried) during storage as depicted in Table 1. The USFAs mainly composed of erucic acid (C22:1), paullinic acid (C20:1), linolenic acid (C18:3), linoleic acid (C18:2), oleic acid (C18:1), and palmitoleic acid (C16:1). Among USFAs, linoleic acid (C18:2) was the most dominant compound. Furthermore, the SFAs consisted of 10 major compounds, including; lignoceric acid (C24:0), tricosylic acid (C23:0), behenic acid (C22:0), arachidic acid (C20:0), stearic acid (C18:0), margaric acid (C17:0), palmitic acid (C16:0), pentadecylic acid (C15:0), myristic acid (C14:0) and lauric acid (C12:0). The C16:0 and C18:0 were the most highly significant compounds of the SFAs. Overall, the concentrations of C18:3, C18:2, C18:1, and C16:0 were higher in raisin. Both drying methods had a different effect on the FA profile during storage. There were 14 FAs that were present in both air-dried and sun-dried raisins. In addition to this, two FAs (C16:1; C20:1) were only identified in air-dried raisins. 

**Figure 1:**
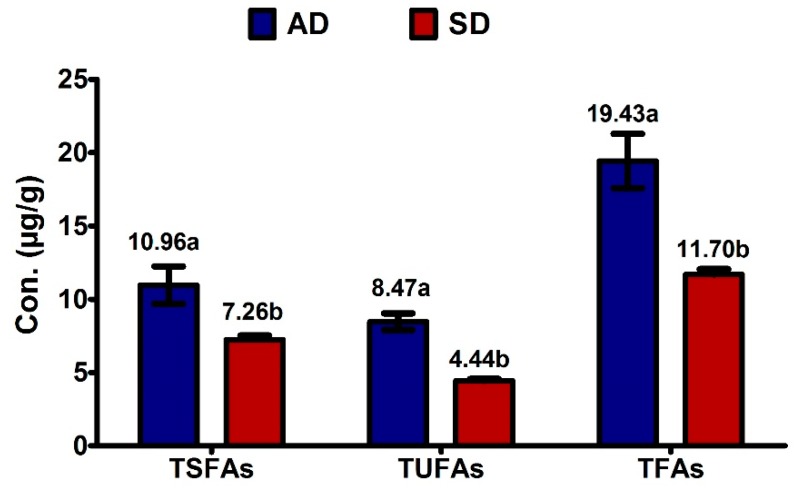
Effect of drying method on total saturated fatty acids (TSFAs), total unsaturated fatty acids (TUSFAs) and total fatty acids (TFAs) of raisins. Different lettering indicates a significance level *p* < 0.005; *n* = 3.

The total concentration of SFAs, USFAs, and total FAs was comparatively higher in those raisins dried by the air as compared to those dried by the sun (Figure 1). The most striking result emerging from the data is that the concentrations of all FAs (SFAs and USFAs) were significantly higher in air-dried raisins than sun-dried raisins (Table 1). The results showed that the concentrations of C16:0, C18:0, and C18:2 were higher in air-dried raisins at the start of storage and then decreased with the time duration, while the C15:0 content increased with storage duration. In contrast, the concentrations of other FAs did not change in storage with respect to drying methods.

### 2.2. Fatty Acid-Derived Volatile Compounds

**Figure 2:**
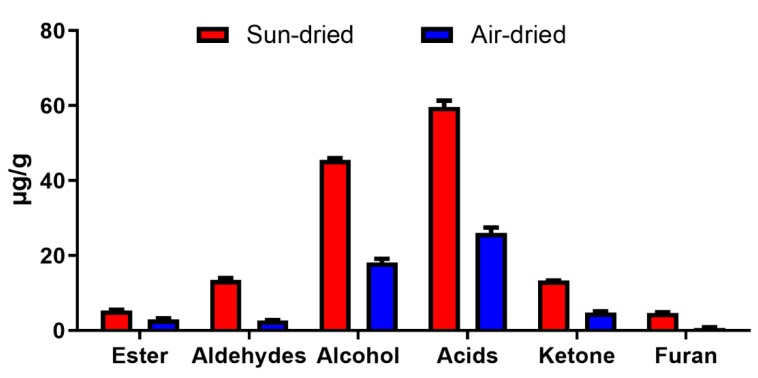
Effect of sun- and air-drying methods on the different classes of fatty acid-derived compounds.

Twenty-six aroma compounds, including 7 aldehydes, 9 alcohols, 4 esters, 4 acids, 1 ketone, and 1 furan, were identified in raisins during storage. Acids were noticed to have the highest content in raisins dried by both methods, followed by alcohol, aldehyde, ketone, furan, and ester. Overall, higher contents were observed in sun-dried raisins (Figure 2).

**Figure 3:**
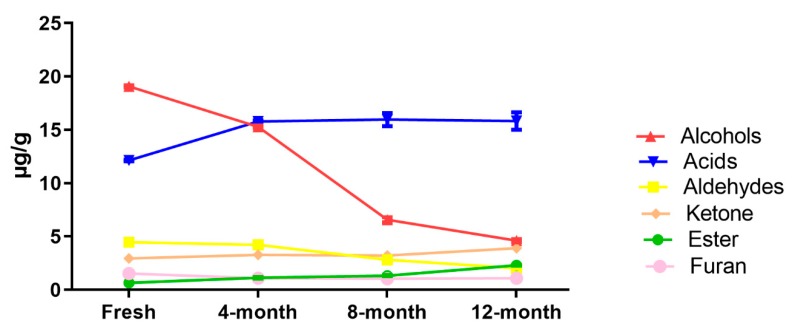
Post-storage changes of different classes of fatty acid-derived compounds in sun-dried raisins.

**Figure 4:**
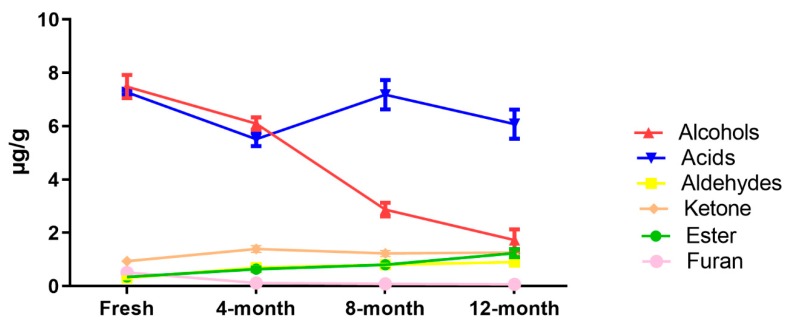
Post-storage changes of different classes of fatty acid-derived compounds in air-dried raisins.

During storage, in sun-dried raisins, the aldehydes, alcohol and furan compound contents decreased as time went by, but the ester and ketone contents increased. The concentrations of main class (acids) were less at the fresh stage compared to at 4, 8 and 12 months of storage, but similar results were found during storage (Figure 3). Furthermore, during storage, as time passed in the air-dried raisins, the concentration of ester and aldehydes increased, but the alcohol and furan concentration decreased. The compounds belonging to the acid and the ketone groups exhibited asymmetrical effects during storage (Figure 4). 

A total number of 26 FA-derived volatile compounds in raisins were listed, among which 20 compounds were identified in both drying methods. Of the remainder, 5 compounds were only detected in sun-dried raisins and 1 compound was only identified in air-dried raisins (Supplementary Table S1). Ethyl octanoate, methyl octanoate, 2-ethyl-1-hexanol, (*E*)-2-heptenal, and (*E*)-2-octen-1-ol were not found in air-dried raisins, while 2-nonanol was not recognized in those raisins that were dried under the sun. A few volatile compounds such as (*Z*)-3-hexen-1-ol and methyl octanoate in sun-dried raisins, and (*E*,*E*)-2,4-heptadienal and decanal in air-dried raisins, were not found in the fresh sample, but were quantified during storage. Furthermore, compounds such as decanal, 1-hexanol, 3-octen-2-one and 2-pentyl furan were the leading compounds on the basis of concentration, and their contents decreased with the passage of storage. On the other hand, the concentrations of heptanoic acid and octanoic acid were directly proportional to storage intervals (Supplementary Table S1).

### 2.3. Effect of Drying Method on Fatty Acids and UFAO-Derived Volatile Compounds

**Figure 5:**
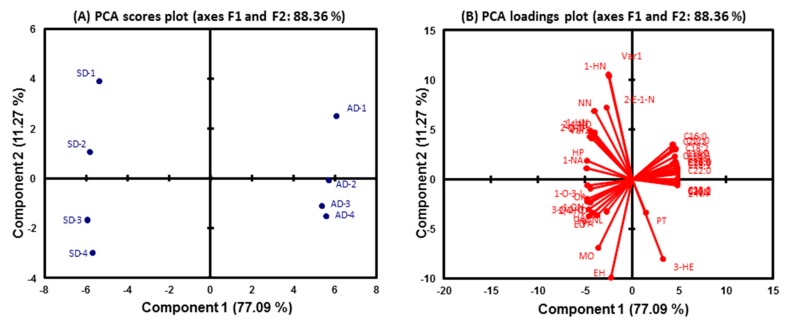
PCA (**A**) score plot for samples and (**B**) loadings plot based on fatty acids and their derived volatile compounds during storage.

During storage, the FA composition and intensity of UFAO-derived volatiles of TS raisins were analyzed using principal component and K-means analysis to determine the effect of the sun- and air-drying methods on raisin FAs and their volatiles. With respect to the drying method (air and sun), the first two principal components (PCs) represented 88.36% of the total variance (Figure 5A). The sun-dried raisins were well separated from the air-dried raisins by PC1. The PC1, which accounted for 77.09% of the total variance, was characterized by all SFAs and USFAs, as well as three VOCs, including pentanol, 2-nonanol and (*Z*)-3-hexen-1-ol (Figure 5B). The remaining UFAO-derived compounds were considered to be the part of belonging to sun-drying methods. 

### 2.4. Hierarchical Cluster Analysis

**Figure 6:**
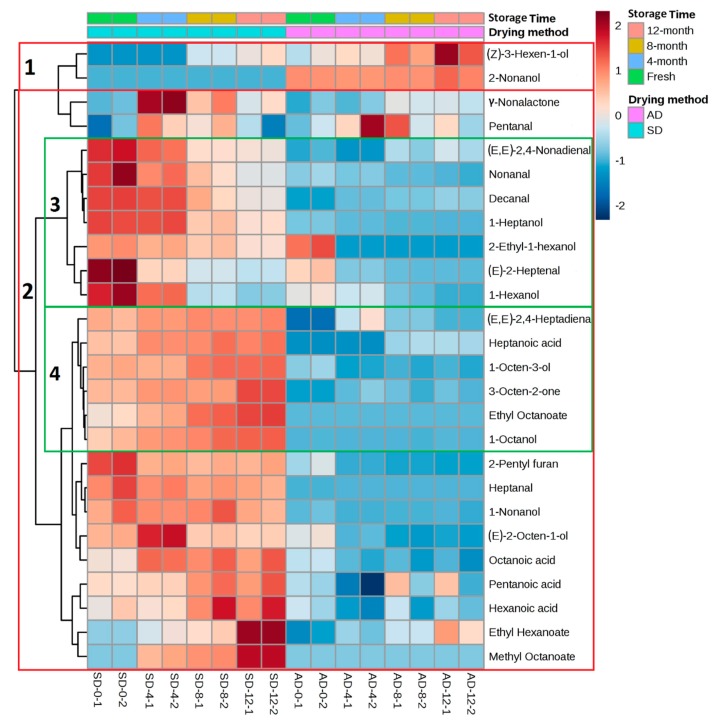
Heatmap visualization of fatty acid-derived volatile compounds of Thompson Seedless raisins.

With respect to air- and sun-drying methods, Figure 6 shows the accumulation pattern of different FA-derived volatile compounds in TS raisins. The cluster of volatile compounds was drawn using hierarchical cluster analysis. The volatile compounds with a similar effect were mainly classified into two main clusters on the basis of the calculated distance. In the air-drying and sun-drying method, higher amounts of volatile compounds were noted in clusters 1 and 2, respectively. Due to the changing trend of volatile compounds during storage, cluster 2 was further divided into two sub-clusters, 3 and 4. Cluster 3 represents those compounds whose concentration decreased during storage, while increasing trends are shown in 4. 

## 3. Discussion

From a nutritional point of view, FAs play a key role in human life. In particular, USFAs have a significant importance for human health, and are responsible for decreasing the cholesterol level, as well as preventing different diseases such as cancer, heart disease, atherosclerosis, and diabetes [16]. Among USFAs, linoleic acid (C18:2) was the most dominant compound, as mentioned in the previous studies with respect to different raisins varieties [17]. As per the results, the number of USFAs, as well as the concentrations of SFAs and USFAs, was significantly higher in the air-dried raisins. The temperature and heat are significantly higher during the sun-drying of raisins as compared to air-drying, and this has been found to be more favorable for the autoxidation reaction [8], and is responsible for the conversion of FAs into their volatile compounds. Regardless of C16:0, C18:0, and C18:2, the concentration of other FAs compounds did not change during the storage of raisins.

With respect to the aroma, FAs are key precursors for the formation of flavor in different fruits [18]. Autoxidation plays a dynamic role in catalyzing USFAs into C6 and C9 volatiles (aldehydes and alcohol), which develop a distinct aroma [19]. Overall, 26 FA-derived compounds were quantified in this study that had already been described in our previous work [4,6,20]. The compounds generated by sun-drying had higher concentration compared to those generated by air-drying. This is due to higher temperature and heat, which can be considered to be favorable for producing a higher concentration of FA-derived volatile compounds [6].

The descriptions, precursors of identified compounds [11,12,21,22,23,24], and aroma descriptors [20,25,26,27] are presented in Table 2. The fruity, green and sweet aromas [25,26] producing compounds such as decanal, 1-hexanol, 3-octen-2-one and 2-pentyl furan were the leading compounds in terms of concentration, and their contents decreased with storage time. In addition, the fatty and cheesy aroma [26]-producing compounds (heptanoic acid and octanoic acid) were considered, and these had a direct relationship with storage. Overall the concentration of fruity, floral and herbaceous aroma-producing compounds was higher throughout the study. Only the amount of (*Z*)-3-hexen-1-ol was higher in air-dried raisins and increased gradually as the storage period progressed, and their precursor had not been reported in the literature. The compounds (*E*)-2-heptenal, (*E*,*E*)-2,4-nonadienal and nonanal were derived from linoleic acids [11,12], while decanal and 1-heptanol came from oleic acids [22,23], and showed higher concentration at fresh stage, with volatility decreasing with the extension of storage duration, which might be due to the loss of their high proportion of volatilization by evaporation [28] in storage. The derivates of oleic acid (1-octanol), linoleic acids (1-octen-3-ol, heptanoic acid, and ethyl octanoate), linolenic acid (*E*,*E*)-2,4-heptadienal) and arachidonic acid (3-octen-2-one) showed lower content in fresh raisins, with their concentration being increased as the storage time increased. In sun-dried raisin, all acidic compounds octanoic acid, heptanoic acid, pentanoic acid, and hexanoic acid showed higher concentrations when specifically stored for 8 and 12 months, but in air-dried raisin, these showed irregular trends during storage. The acid compounds were derived from the autoxidation of methyl linoleic acid [24]. Some volatile compounds are specific to the drying method, such as 2-nonanol and methyl octanoate, which were only present in air-dried and sun-dried raisins, respectively. These showed higher concentration at 12 months of storage. The only furan compound, ‘2-pentyl furan’, came from linoleic acid [11,23] and had a higher concentration in fresh raisin as compared to storage for both drying methods. These results coincided with our previous report on changes of volatile compounds in pre-treated raisins during storage [4].

## 4. Materials and Methods 

TS grapevine was planted in a commercial orchard located in Turpan (at northern hemisphere with latitude 42.948 and longitude 89.179 coordinates) Xinjiang province, China. The specific air-drying method reported by our lab [6] and common sun-drying methods were used for dehydrating the fully ripe grapes (TSS > 20 °Brix), which required 42 and 28 days, respectively. The drying process continued until the weight of TS raisins remained unchangeable, up to a maximum of 3 days, and the moisture contents were less than 15%. Then, 1 kg raisins samples from each drying method (sun and air) were packed into plastic bags and preserved at room temperature (24 ± 2 °C) for 12 months (October 2014 to September 2015). The post-storage changes of TSS (°Brix), pH, and moisture content (%) of the sun- and air-dried raisins are shown in Supplementary Figure S1. The FAs and their derived volatile compounds were detected and identified after 4-month intervals of storage (i.e., at 0, 4, 8 and 12 months). All of the samples were kept in the freezer (at −40 °C) until use, and each one was analyzed in triplicate.

### 4.1. Analysis of Fatty Acids

#### 4.1.1. Sample Preparation

The extraction of FA was conducted as in our earlier study [29], with minor modifications. Firstly, raisins were deep-frozen using liquid nitrogen with added quartz sand (1:1), and then immediately pulverized by an electronic shaker. 

#### 4.1.2. Extraction and Methylation

A two-gram raisin sample was added to a 50 mL glass flask with 25 mL *n*-hexane and 8 mL methanol as the extraction solvent. Prior to extraction, the solvent was shaken by an electronic oscillator for 30 min at 28 °C and the residue was collected in a trice using the same procedure. The collected organic layers were immediately put into a vacuum rotary evaporator (30 °C) for drying. After evaporation of the solvent, 5 mL of 1% H_2_SO_4_/methanol (*w*/*v*) solution was added to methylate the lipid extracts for 2 h at 65 °C and then cooled down on room temperature. Afterward, *n*-hexane (3 mL) and distilled water (3 mL) were added to separate the methyl ester of FA from this two-phase mixture, and this was repeated in triplicate. The hexane layer was collected and dried under a gentle stream of nitrogen. Finally, 990 µL *n*-hexane and 10 µL methyl nonadecanoate (0.04 mg/mL) were added, and filtered through the organic phase microfiltration membrane (0.22 μm) and then immediately analyzed by gas chromatography (GC) coupled with a 5975 mass spectrum (MS). 

#### 4.1.3. GC-MS Condition

The FA methyl esters were detected by an Agilent GC (7890, (J&W Scientific, Folsom, CA, USA)) equipped with MS (5975) (J&W Scientific, Folsom, CA, USA). A capillary column 0.25 µm in thickness (60 m × 0.25 mm id HP-INNOWAX) (J&W Scientific, Folsom, CA, USA) was used to determine the FA. In this experiment, the temperature condition of GC-MS was quite different from our previous work [29]. Initially, the flow rate of helium (carrier gas) was 1 mL/min, and the oven temperature was 40 °C for 1 min, elevated to 220 °C at 25 °C/min, and then changed to 250 °C at 5 °C/min. In the end, the temperature was held for 20 min at 250 °C. The temperatures of the injector and transfer line were maintained at 250 °C and 280 °C, respectively.

### 4.2. Fatty Acid-Derived Volatile Compounds

#### 4.2.1. Sample Preparation

The raisin samples for the extraction of FA-derived volatile compounds were prepared according to precedent [6]. The slurries were prepared by soaking frozen raisins in an equal weight of distilled water and stored overnight at 4 °C. On the very next day, the samples were thoroughly homogenized and macerated (4 h). The pulp was immediately centrifuged (at 8000 rpm; 15 min; 4 °C) and the whole supernatant was obtained thereafter. The clear supernatant was used for the identification of free-form VOCs. 

#### 4.2.2. SPME Condition

For extraction, the clear supernatant (5 mL) and 4-methyl-2-pentanol (10 µL; 1.0018 mg/L; as an internal standard) were blended with a magnetic stirrer in a vial (15 mL). NaCl (1.3 g) was mixed in, and the vial was tightly capped with a stopper (PTFE-silicon) (CNW Technologies, Dusseldorf, Germany). The sample-containing vial was then equilibrated (40 min; 60 °C) on a heating stand. The extraction was executed by inserting solid-phase micro-extraction fiber (CAR/PDMS/DVB) (Supleco, Darmstadt, Germany) into the headspace over a period of 40 min, with constant heating and agitation. At the end of the extraction of volatiles, the fiber was instantly desorbed for 8 min into the GC injection port.

#### 4.2.3. GC-MS Condition 

The detection of FA-derived compounds was accomplished on the same GC-MS (mentioned earlier), but the oven temperature was changed, and was automated as follows: temperature and time: 50 °C for 1 min, and increased to 220 °C at a rate of 3 °C/min and then maintained at 220 °C for 5 min. Mass spectra were accomplished using the electron impact (EI) approach by obtaining the ionization energy (IE, 70 eV) and source temperature (230 °C). The full-scan approach was applied along with mass range (*m*/*z*; 20–450), and then further action took place in a selective ion manner under auto-tune conditions.

ChemStation software (Agilent Technologies, Santa Clara, CA, USA) was used for managing and investigating the data. The identification of compounds was carried out using retention indices (RI) of reference standards, while the mass spectra were matched with the NIST 08 (National Institute of Standards and Technologies, Gaithersburg, MD, USA) library. Wherever the reference standard was not available, tentative identification was performed by comparing the mass spectrum of the NIST library and RI that was cited in the literature.

#### 4.2.4. Quantification Method

The quantification method was standardized in our previous work [6], with minor modifications. A simulated solution was prepared by taking the average concentration of the sugar and acids in the raisin supernatant. Then, the solution was prepared in distilled water containing glucose (400 g/L) and tartaric acid (5 g/L). The pH of the solution was acclimatized to 4.2 with 1 M solution of NaOH. Now, the known concentrations of standard compounds were mixed in ethanol (HPLC-grade) and diluted up to the fifteenth level using the simulated raisin solution. Each level was extracted and analyzed in a similar manner as that of the raisin supernatant. Furthermore, the volatile compounds that did not fulfill chemical standards were juxtaposed with those standards that had identical functional groups/similar numbers of C-atoms. In the end, the content of identified volatile compounds was computed by the characteristic ion peak area with regards to the IS.

### 4.3. Statistical Analysis

Heatmap cluster analysis (HCA)and principal component analysis (PCA) were performed based on the concentration of identified volatile compounds and FAs by the Metabo-Analyst 3.0 software (http://www.metaboanalyst.ca/) through time series analysis. Auto-scaling (mean-centered and divided by the standard deviation of each variable) was used to normalize the data. A two-way statistical analysis of variance was utilized to calculate the influence of drying techniques during the storage time on different classes of volatile compounds employing a significance level *p* < 0.005. 

## 5. Conclusions

Raisins were prepared from TS grapes using air- and sun-drying techniques. The dried raisins were stored for 0, 4, 8 and 12 months at room temperature and the FAs were then identified and quantified along with their derived compounds under GC/MS. A total of 16 and 14 FAs were identified in air-dried and sun-dried raisins, respectively. Paullinic acid (C20:1) and palmitoleic acid (C16:1) were only identified in air-dried raisins. Overall, a higher content was seen in air-dried raisins than sun-dried raisins, specifically at the fresh stage. Generally, non-significant changing effects of FAs were seen during storage. The concentrations of FA-derived raisin compounds were significantly higher in sun-dried raisins. The compounds (*E*)-2-heptenal, (*E*,*E*)-2,4-nonadienal, nonanal, 1-hexanol, 2-ethyl-1-hexanol, decanal, 1-heptanol were higher in fresh raisins, while ester compounds (ethyl hexanoate, ethyl octanoate, and methyl octanoate) and ketone compound (3-octen-2-one) were higher after a storage period of 12 months. The outcomes of this research indicate that the air-drying method improves the nutritional quality in terms of FA, and the sun-dried method enhances the flavor of TS raisins.

## Figures and Tables

**Table 1:** Identification of different fatty acids (saturated and unsaturated) in air-dried and sun-dried raisins during storage.

S/No	RT	Fatty Acids	Common Name	ID	Linear Equation	R	Air-Dried				Sun-Dried			
Saturated Fatty Acid							0-M	4-M	8-M	12-M	0-M	4-M	8-M	12-M
1	10.4	C12:0	Lauric acid	1	y = 0.1282x + 0.4898	0.999	2.39a	0.56b	0.56b	0.56b	0.51b	0.49b	0.50b	0.50b
2	11.8	C14:0	Myristic acid	1	y = 0.1344x + 0.919	0.992	1.16a	1.07b	1.09b	1.09b	0.99c	0.95d	0.95d	0.94d
3	12.6	C15:0	Pentadecylic acid	1	y = 0.1395x + 1.8572	0.991	1.89b	1.90b	1.91a	1.91a	1.88c	1.86d	1.87d	1.86d
4	13.4	C16:0	Palmitic acid	1	y = 0.0148x + 0.8343	0.990	1.68a	1.37b	1.26c	1.27c	0.97d	0.91e	0.91e	0.92e
5	14.4	C17:0	Margaric acid	1	y = 0.1428x + 0.5216	0.993	0.63a	0.62a	0.62a	0.62a	0.55b	0.53b	0.54b	0.53b
6	15.4	C18:0	Stearic acid	1	y = 0.1428x + 0.5216	0.993	4.41a	3.75b	3.45c	3.38c	1.14d	1.10d	1.04d	1.10d
7	18.2	C20:0	Arachidic acid	1	y = 0.2098x + 0.6079	0.991	0.99a	0.96a	0.95a	0.96a	0.69b	0.65b	0.67b	0.66b
8	21.9	C22:0	Behenic acid	1	y = 0.1511x + 0.2175	0.995	0.70a	0.68a	0.69a	0.68a	0.29b	0.27b	0.28b	0.28b
9	24.4	C23:0	Tricosylic acid	1	y = 0.1259x + 0.2172	0.995	0.28ab	0.28ab	0.29a	0.29a	0.23c	0.23c	0.23c	0.23c
10	27.6	C24:0	Lignoceric acid	1	y = 0.0953x + 0.1182	0.997	0.26a	0.24a	0.24a	0.24a	0.15b	0.14b	0.14b	0.14b
**Unsaturated Fatty Acid**							**0-M**	**4-M**	**8-M**	**12-M**	**0-M**	**4-M**	**8-M**	**12-M**
11	13.8	C16:1	Palmitoleic acid	1	y = 0.1042x + 0.7286	0.991	0.73a	0.74a	0.74a	0.74a	NF	NF	NF	NF
12	15.8	C18:1	Oleic acid	1	y = 0.1283x + 1.5322	0.993	2.03a	1.51b	1.58b	1.62b	0.70c	0.66d	0.64d	0.66d
13	16.4	C18:2	Linoleic acid	1	y = 0.0883x + 1.3784	0.993	3.52a	2.78b	2.66bc	2.53c	1.61d	1.47d	1.50d	1.52d
14	17.3	C18:3	Linolenic acid	1	y = 0.1006x + 0.5955	0.997	1.96a	1.93ab	1.90b	1.92ab	1.59c	1.57c	1.57c	1.59c
15	18.6	C20:1	Paullinic acid	1	y = 0.053x + 0.2264	0.994	0.24a	0.24a	0.24a	0.24a	NF	NF	NF	NF
16	22.4	C22:1	Erucic acid	2			1.54a	1.10b	1.04b	1.10b	0.68c	0.63c	0.67c	0.66c

RT: retention time. ID (identification method): 1, identified, mass spectrum and RI were in accordance with standards; 2, tentatively identified, mass spectrum matched in the standard NIST 2008 library and RI matched with NIST Standard Reference Database (NIST Chemistry WebBook). Linear equation: concentration in mg/L; x, peak area ratio of a compound into the internal standard (4-methyl-2-pentanol). R: Regression coefficient.

**Table 2:** The description of identified UFAO-derived compounds in Thompson seedless raisins.

S/No	RI	Volatile Compounds	Source	ID-M	Ion (*m*/*z*)	Formula	Precursor	Aroma Descriptor
1	1227	Ethyl hexanoate	Ester	1	88	C_8_H_16_O_2_	Linoleic acid **^F^**	Fruity, apple-like **^e^**
2	1432	Ethyl octanoate	Ester	2	74	C_10_H_20_O_2_	Linoleic acid, Linolenic acid **^F^**	Apple, fruity, sweet **^e^**
3	1378	Methyl octanoate	Ester	2	88	C_9_H_18_O_2_	Oleic acid **^A^**	Fruity, citrus like **^e^**
4	2035	γ-Nonalactone	Ester	2	85	C_9_H_16_O_2_	Linoleic acid ^**F**^	Coconut, peach **^e^**
5	975	Pentanal	Aldehyde	2	44	C_5_H_10_O	Linoleic acid, Arachidonic acid **^E^**	Fat, Green **^e^**
6	1178	Heptanal	Aldehyde	2	44	C_7_H_14_O	Linoleic acid **^D,E^**, Oleic acid **^E^**	Dry fish, solvent, smoky **^e^**
7	1325	*(E)*-2-Heptenal	Aldehyde	2	41	C_7_H_12_O	Linoleic acid **^D,E^**	Fatty, soapy, tallow **^e^**
8	1393	Nonanal	Aldehyde	1	57	C_9_H_18_O	Linoleic acid **^B^**	Green, Fruity **^e^**
9	1501	Decanal	Aldehyde	1	43	C_10_H_20_O	Oleic acid **^D,E^**	Sweet, citrus, green **^c^**
10	1497	*(E,E)*-2,4-Heptadienal	Aldehyde	2	81	C_7_H_10_O	Linolenic acid **^E^**	Fatty, hay **^a^**
11	1705	*(E,E)*-2,4-Nonadienal	Aldehyde	2	81	C_9_H_14_O	Linoleic acid **^D,E^**	Fatty, oily **^a^**
12	1349	1-Hexanol	Alcohol	2	56	C_6_H_14_O	NF	green **^a^**
13	1395	*(Z)*-3-Hexen-1-ol	Alcohol	2	67	C_6_H_12_O	NF	Fruity, green **^a^**
14	1449	1-Octen-3-ol	Alcohol	1	57	C_8_H_16_O	Arachidonic acid **^D^,** Linoleic acid **^A^**	Mushroom, fruity **^e^**
15	1453	1-Heptanol	Alcohol	2	70	C_7_H_16_O	Oleic acid **^A^**	Grape, sweet **^b^**
16	1487	2-Ethyl-1-hexanol	Alcohol	1	57	C_8_H_18_O	NF	Floral, sweet fruity **^b^**
17	1555	1-Octanol	Alcohol	1	56	C_8_H_18_O	Methyl Oleate **^B^**	Citrus, rose **^c^**
18	1614	*(E)*-2-Octen-1-ol	Alcohol	2	57	C_8_H_16_O	Oleic acid **^A^**	Fatty, rancid **^a^**
19	1657	1-Nonanol	Alcohol	1	56	C_9_H_20_O	NF	Floral **^a^**
20	1488	2-Nonanol	Alcohol	2	45	C_9_H_20_O	NF	NF
21	1740	Pentanoic acid	Acid	2	60	C_5_H_10_O_2_	Methyl Linoleic acid **^C^**	Sweet **^a^**
22	1847	Hexanoic acid	Acid	1	60	C_6_H_12_O_2_	Methyl Linoleic acid **^C^**	Rancid, Cheese, Fatty **^e^**
23	1953	Heptanoic acid	Acid	1	60	C_7_H_14_O_2_	Methyl Linoleic acid **^C^**	Sweety, cheesy **^d^**
24	2060	Octanoic acid	Acid	1	60	C_8_H_16_O_2_	Methyl Linoleic acid **^C^**	Rancid, Cheese, Fatty **^e^**
25	1416	3-Octen-2-one	Ketone	2	55	C_8_H_14_O	Arachidonic acid **^E^**	Green, fruity **^a^**
26	1224	2-Pentyl furan	Furan	2	81	C_9_H_14_O	Linoleic acid **^B,E^**	Fruity, green, sweet **^e^**

Reported the precursor (fatty acids) of their respected volatile compounds (**^A^** Frankel 1980; **^B^** Frankel, Neff, and Edward S. 1981; **^C^** Horvat et al. 1968; **^D^** Meeting, Ho, and Hartman 1994; **^E^** Whitfield and Mottram 1992; **^F^** Belitz, Garcia and Wayne, 2004); NF = not found; Retention Indices (RI): Kovats RI was calculated based on the *n*-alkane series (C6–C24) on the poly (ethylene glycol) (PEG) column under the same chromatographic conditions. Aroma descriptors were obtained from “Flavornet and human odor space” the LRI and odor database (**^a^** http://www.odour.org.uk/odour/index.html) and from reported literature (**^b^** Jiang & Zhang, 2010; **^c^** Wang et al., 2017; **^d^** Welke et al., 2014; **^e^** Wu et al., 2016). Identification method (ID-M): 1, identified, mass spectrum and RI were in accordance with standards; 2, tentatively identified, mass spectrum matched in the standard NIST 2008 library and RI matched with NIST Standard Reference Database (NIST Chemistry WebBook). Ion (*m*/*z*): The characteristic ion (*m*/*z*) was used for choosing the corresponding compound and evaluating the peak areas of them in order to avoid possible interference by other compounds.

## Supplementary Materials

The following are available online: Figure S1: Effect of drying method on TSS, pH and moisture content of stored raisins, Table S1: Effect of drying methods on UFAO-derived compounds of Thompson Seedless raisins during storage.

## Abbreviations

USFAs	Unsaturated Fatty Acids
SFAs	Saturated Fatty Acids
FAs	Fatty Acids
TS	Thompson Seedless
GC/MS	Gas Chromatography/Mass Spectrometry
